# Biomechanical Evolution in Longitudinal Analysis of Lumbar Stress Injuries in Fast Bowlers: A Systematic Review

**DOI:** 10.7759/cureus.99363

**Published:** 2025-12-16

**Authors:** C Arun, Sai Aditya Raman, Nimishaanth SS, Thiagarajan KA, Arumugam Sivaraman

**Affiliations:** 1 Faculty of Sports and Exercise Science, Sri Ramachandra Institute of Higher Education and Research, Chennai, IND; 2 Department of Arthroscopy and Sports Medicine, Sri Ramachandra Institute of Higher Education and Research, Chennai, IND

**Keywords:** cricket fast bowling injuries, fast bowling biomechanics, injury prevention and rehabilitation, key injury predictors, key performance indicators, lumbar stress injuries, performance indicator analyst, sports biomechanics, systematic review

## Abstract

Fast bowling involves a highly complex and physically demanding motion sequence, placing fast bowlers under substantially greater workloads than other cricketing roles. Consequently, they experience a disproportionately high incidence of injuries, particularly to the lower back, with lumbar stress fractures being especially common in adolescent bowlers. These injuries are multifactorial, arising from repeated exposure to high spinal loads, underscoring the need for comprehensive biomechanical analysis and targeted preventive strategies to enhance safety and prolong athletic careers. This systematic review synthesizes 15 years (2008-2023) of research to identify injury patterns and biomechanical contributors, aiming to inform safer, sustainable training practices. A comprehensive search of MEDLINE, Embase, SPORTDiscus, CINAHL, Scopus, Web of Science, PubMed, and Google Scholar identified 21,550 records. After screening 21,406 titles and abstracts, 517 reports were sought for retrieval, and 491 were assessed for eligibility. Ultimately, 37 studies met the inclusion criteria. Eligible studies included prospective and retrospective cohort designs, analytical cross-sectional studies, and randomized controlled trials. Only peer-reviewed, full-text, English-language publications were considered, while case reports, reviews, and non-peer-reviewed articles were excluded. Screening and verification were conducted using RAYYAN and Mendeley, with an independent multi-reviewer assessment to ensure rigor. Findings highlight that shoulder counter-rotation exceeding 25°, trunk lateral flexion, and reduced knee flexion (<30°) at front-foot contact significantly elevate lumbar and lower-limb loading. Poor lumbopelvic control increases lumbar stress injury risk by up to 88%, whereas greater ankle dorsiflexion (>30°) demonstrates a protective effect via improved shock absorption. Preventive strategies should prioritize early biomechanical screening, workload regulation, and targeted lumbopelvic strengthening. Rehabilitation approaches must emphasize gradual workload progression and refined movement control, supported by long-term biomechanical monitoring to enhance performance and minimize injury risk.

## Introduction and background

Cricket is globally recognized as the second most widely played sport, requiring significant skill and endurance, particularly for fast bowlers, whose role involves delivering high-speed balls to challenge batters [1,2]. The biomechanics of fast bowling includes a complex motion sequence where linear momentum from the run-up is converted into angular momentum during the delivery phase, highlighting the physical and technical demands of this role [2,3].

Fast bowlers experience greater physical workloads and activity levels compared to other cricketing roles, leading to a higher incidence of injuries, particularly in the lower back [4-8]. Lower back injuries, including lumbar stress fractures, are prevalent, especially in adolescent fast bowlers, with reported injury rates ranging from 11% to 67% [9,10]. These injuries are multifactorial, necessitating comprehensive analysis to identify contributing factors and devise effective prevention strategies [11,12].

Biomechanical factors, such as contralateral trunk side flexion and high vertical ground reaction forces often exceeding six times the body weight, are significant contributors to lumbar spine stress during bowling [2,3,13]. Repeated exposure to these forces can lead to stress-related injuries, emphasizing the need for targeted interventions to enhance safety and prolong careers.

The study aims to analyze 15 years (2008-2023) of biomechanical data on fast bowling injuries, focusing on lumbar stress injury patterns. By integrating historical and contemporary insights, it seeks to improve players’ safety and advance sustainable practices in cricket.

## Review

Methodology

This study was approved by the Institutional Ethical Committee, Sri Ramachandra Institute of Higher Education and Research (SRIHER) (reference number: SRIHER2025/PGMC522/CSS). The manuscript titled “Biomechanical Evaluation in Longitudinal Analysis of Lumbar Stress Injuries in Fast Bowlers” was reviewed and approved for submission by the Publications Guidelines and Monitoring Committee (PGMC).

This systematic review examined studies on lumbopelvic injuries in cricket fast bowlers using advanced biomechanical assessment tools such as three-dimensional (3D) motion capture and force plates to identify injury mechanisms and associated risk factors. Studies reporting injury prevalence, incidence, and influences of workload, gender, and bowling frequency were included, while pilot studies, case-control designs, conference papers without peer review, review articles, and datasets from published research articles before 2008 were not included in the study to maintain rigor.

The review incorporated prospective and retrospective cohort studies, analytical cross-sectional studies, and randomized controlled trials to provide robust evidence on injury patterns and their determinants. Only peer-reviewed, full-text articles published in the English language from 2008 to 2023 were selected, ensuring high-quality and accessible data for analysis. The search strategy employed Boolean operators to refine results across seven databases, including MEDLINE, Embase, SPORTDiscus, CINAHL, Scopus, Web of Science, PubMed, and Google Scholar. Keywords targeted fast bowlers’ injuries, lumbar and lumbopelvic injuries, and workload-related factors. Inclusion criteria prioritized studies on cricket fast bowlers across all skill levels, formats, and ages, while poorly defined, non-peer-reviewed, or unavailable studies were excluded.

**Figure 1:**
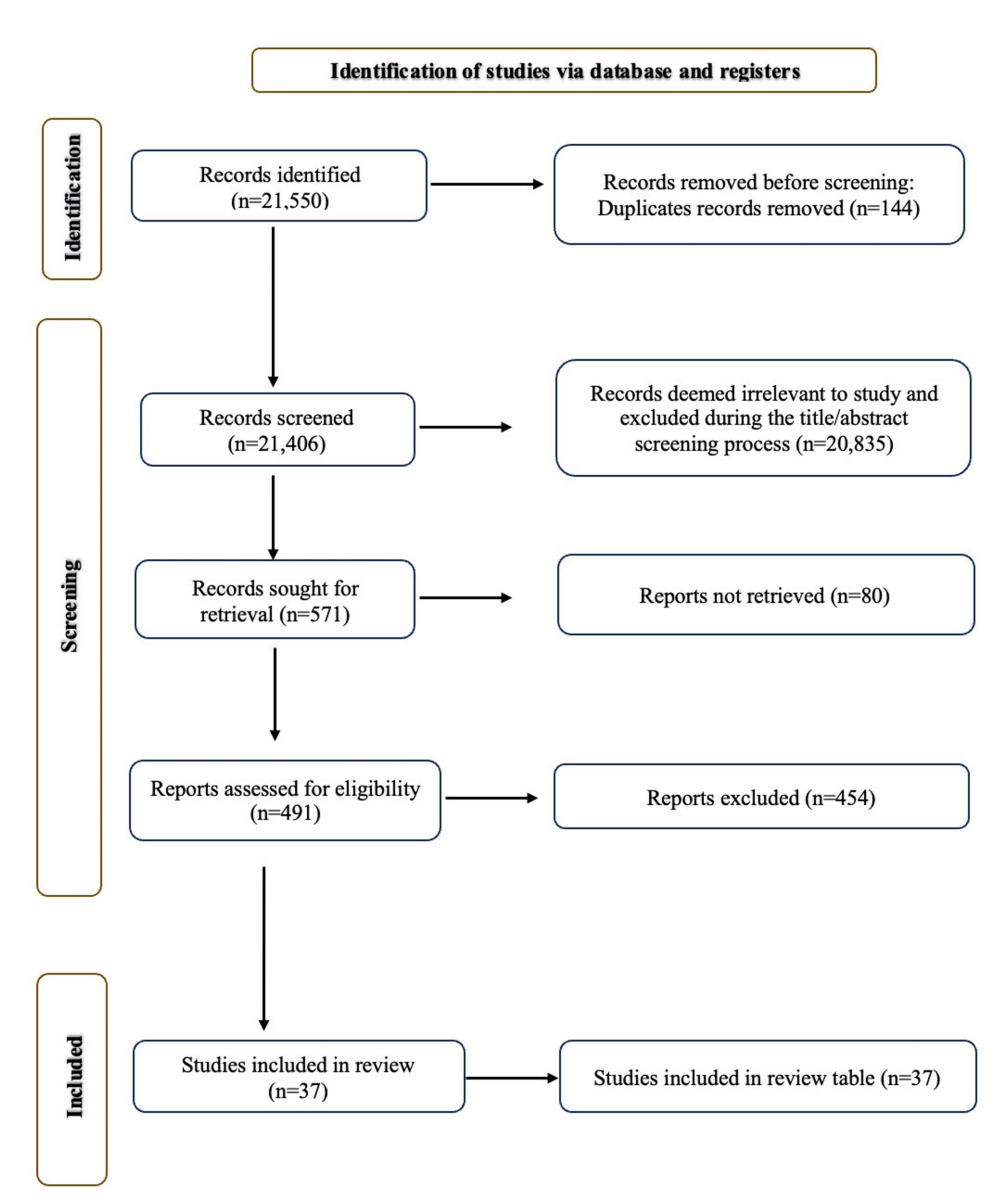
Preferred Reporting Items for Systematic Reviews and Meta-Analyses (PRISMA) flowchart for literature review.

Screening methods involved tools such as RAYYAN for initial filtering and duplicate removal and Mendeley for final verification, supported by a multi-reviewer assessment to ensure reliability. Risk of bias was not evaluated, as the review aimed to summarize methodologies for analyzing workload and biomechanical factors in cricket, rather than critiquing study outcomes. The systematic review process was conducted and reported in accordance with the Preferred Reporting Items for Systematic Reviews and Meta-Analyses (PRISMA) 2020 guidelines [14], ensuring transparency and methodological rigor in the identification, screening, and inclusion of studies (Figure 1).

Three reviewers (AN, NS, and KAT) independently screened all titles and abstracts according to the eligibility criteria. Full-text articles of potentially relevant studies were then retrieved and assessed in detail for inclusion. Any disagreements were resolved through discussion or, when necessary, arbitration by the lead reviewer (SAR) and (AMSN), who also supervised the final inclusion decisions and the data synthesis process.

Results

This review synthesizes findings (Table 1) from 37 studies (2008-2023) on lumbar stress injuries in fast bowlers, covering injury epidemiology, biomechanical risks, workload impact, and skeletal adaptations. Key insights reveal injury prevalence, contributing factors, and positional differences.

**Table 1:** Summary of included studies (data synthesized from 2008–2023), presenting study details, design, participant characteristics (age and level of play), study focus, risk factors analyzed, and key findings. BMD = bone mineral density; ROM = range of motion; LBSI = lumbar stress injury; GRF = ground reaction force; LBP = low back pain

Study	Study design	Participants (age, level of play)	Study focus	Risk factor analyzed	Study results
Bansal et al., 2023 [15]	Prospective cohort study	18 elite English female cricketers (mean age: 23.7 ± 4.1 years) and 12 Indian state-level female players (mean age: 18.9 ± 5.1 years)	Comparison of injury patterns, frequency, and risk factors between English and Indian female cricketers	Injury onset mechanism (insidious vs. sudden onset), dominant skill category (batters vs. bowlers), match vs. training injuries, and injury type (muscle, ligament, bone)	English players had 95 injuries (55.8% time-loss), while Indian players had 16 (~58% time-loss). Injury incidence was higher in T20s (310.6/10,000 hours) than ODIs (90.7/10,000 hrs). Common injury sites: wrist/hand (18.9%) and thigh (13.7%) for England; knee and wrist/hand (~33%) for India. English bowlers had more lumbar spine injuries, while Indian batters had the highest injury rates. Most injuries had an insidious onset, linked to bowling, running, and throwing. Serious injuries (31.6%) required 29 days to 6 months of recovery. More injuries occurred in season two (55.8%) than in season one (44.2%)
Always et al., 2023 [16]	Cross-sectional study	Female elite cricketers (national senior and development squads)	Investigated BMD differences by playing position and lumbar/hip asymmetry	Bowling workload, menstrual function, body composition, and bone loading	Fast bowlers had higher lumbar BMD on the contralateral side, while total BMD showed no positional differences. Overall, high BMD in cricketers resulted from impact forces in batting and bowling
Felton et al., 2023 [17]	Cross-sectional biomechanical analysis	45 elite male fast bowlers	Examined ROM constraints in relation to performance and injury risk	Shoulder, hip rotation, and ankle dorsiflexion were analyzed for ball speed and injury risk	ROM measures did not correlate with ball speed, but shoulder and hip ROM influenced technique, performance, and injury risk. Greater ankle dorsiflexion lowered lumbar stress injury risk, while bowling-arm adaptations suggested effects from throwing
Keylock et al., 2023 [18]	Longitudinal study	91 male fast bowlers (14–24 years) and 43 controls from ECB teams, county clubs, and cricket-focused schools	Investigated age-related lumbar spine BMC and BMD development in fast bowlers vs. controls, focusing on asymmetrical adaptation	Examined asymmetrical lumbar adaptation, bone accrual rate, workload impact, and early adaptation injury risks	Fast bowlers showed greater lumbar BMC and BMD growth than controls (55% vs. 41%), with pronounced asymmetry at L3-L4. Peak bone accrual occurred earlier, highlighting the osteogenic benefits of fast bowling but also the need for workload management to reduce injury risks in developing athletes
Walter et al., 2022 [19]	Retrospective surveillance study.	35 first-class male fast bowlers (26 ± 4 years) from New Zealand’s 2017–2018 season	Identified injury prevalence, types, and affected regions in elite fast bowlers	Bowling style, biomechanics, workload, training maladaptations, recurrent injuries, and joint anatomy	66% of players reported injuries, with lower back (19%), ankle/foot, thigh, and shoulder most affected. Acute injuries (68%) were more common than overuse (32%). Lower back injuries caused the highest time loss (691 days), with stress fractures (11%), hamstring strains (9%), and rotator cuff inflammation (9%) being frequent. Injuries occurred mainly while bowling (50%) and during matches (51%)
Keylock et al., 2022 [20]	Prospective cohort study	40 adolescent male fast bowlers (15.5 ± 1.1 years) from academies, schools, and clubs	Examined LBSI incidence, prevalence, and risk factors in adolescent fast bowlers	Age, BMD, flexibility, workload, and movement control	High LBSI incidence (27%); risk linked to skeletal immaturity, high workload, and delayed maturation
Lucente et al., 2021 [21]	Retrospective cohort study	177 elite Australian fast bowlers (107 male, 70 female) from senior state and national levels. Injured bowlers were younger (21.5 years) than non-injured (26.4 years)	Identified risk factors for LBSI in elite fast bowlers, focusing on technique, musculoskeletal traits, and bowling frequency	Excessive counter-rotation, lateral flexion, extended knee, high bowling frequency, and younger age increased LBSI risk, with prior injury as the strongest predictor. No significant musculoskeletal differences	LBSI occurred in 81% of cases contralateral to the bowling arm, mostly at L4 (42%) and L5 (33%). Recurrence was high (63%), with a median return time of 161 days. Injured females bowled more in the last four weeks, while injured males had lower bowling frequency over 12–52 weeks. Key predictors of LBSI were younger age, previous injury, and recent high bowling frequency, explaining 45% of injury risk variance
Dovbysh et al., 2021 [22]	Retrospective epidemiological database analysis	Elite male New Zealand cricketers (2009–2015); Domestic: 17–40 years (28.2); International: 18–38 years (28.9)	Examined injury incidence and prevalence in New Zealand T20 cricket	Injury rates by format, role, and body region; lumbar spine and lower limb injuries most common	T20 had the highest injury rates (domestic: 74.5; international: 144.2/10,000 hours). Fast bowlers (54.3%) were the most injured, spinners the least injured (29.9%). Lumbar spine caused most days lost in domestic players, groin in internationals. T20 injuries increased since 2002–2008
Alway et al., 2021 [23]	Prospective cohort study	50 elite male fast bowlers	Comparing kinematics and kinetics between injured and non-injured bowlers	Rear knee/hip angles, thoracolumbar motion, front hip angle, pelvic tilt, and lumbopelvic control across bowling phases	LBSI occurred in 39 of 50 bowlers. Poor lumbopelvic control and altered motion increased injury risk. A logistic model (rear hip and lumbopelvic angles) predicted 88% of cases, aiding early detection and rehab strategies
Sims et al., 2021 [24]	Retrospective cohort study	222 high-level youth male fast bowlers (age: 17.4 ± 1.1 years)	Examined age, anthropometry, musculoskeletal screening, fitness, bowling volume, and technique as LBSI risk factors	Age, height, bowling speed, and key physical/technical attributes	49 bowlers sustained LBSI. Younger age, taller height, and faster bowling speed were significant risk factors. Injury risk increased 2.99 times per year younger, 1.1 times per cm taller, and 1.1 times per km/hour faster bowling speed. Other unidentified factors likely contribute to LBSI development
Perrett et al., 2020 [25]	Cross-sectional	11 pre-elite and elite fast bowlers, injury-free from lumbar stress fractures and disc herniations for two years	Analyzed intra- and inter-individual variability in spinal kinematics during fast bowling	Examined spinal kinematic variability, its link to fatigue, and its impact on bowling speed	Inter-individual variability in axial rotation and lateral bending was higher than intra-individual. Lateral bending varied between sessions, but axial rotation remained stable. No clear link to ball speed or fatigue, emphasizing the need for individualized assessments
Badhyal et al., 2020 [26]	Experimental study using	Four Indian male state-level fast bowlers (age: 19.50 ± 2.52 years)	Examined lower limb muscle loading in fast bowling using computational biomechanics	Analyzed GRFs, muscle activation, and joint loading during front-foot contact	Identified individual variability in muscle activation and loading, aiding workload management, injury prevention, and rehabilitation
Rao et al., 2020 [27]	Injury surveillance study	319 male cricket players from five State Cricket Associations, aged 18–24 years. Competitive/state-level cricket	Epidemiology of annual musculoskeletal injuries and their risk factors	Injury prevalence, anatomical injury sites, player position, seasonal trends, and loss of playdays	Annual injury prevalence: 10.97%. Most affected areas: shoulder (22.85%), lumbar spine (17.14%), and knee (11.42%). Medium pacers had the highest injury rate (25.71%). Overuse injuries (37.14%) were common in ages 18–24, with 71.42% affecting the lumbar spine. Lumbar spine injuries caused the most lost playdays (34.64%), with bowling injuries accounting for 49.5%. Injuries peaked in December (20%). Four surgeries (11.42%) were reported
Goggins et al., 2020 [28]	Prospective cohort	Elite male first-class, one-day, and T20 cricketers from England and Wales	Injury incidence rates and epidemiology across different formats	Injury rates by format, role-specific prevalence, common injury sites, and seasonal trends	One-day cricket had the highest injury incidence (254/1,000 days), followed by T20 (136/1,000) and first-class (68/1,000). Bowling had the highest injury rate (41.6/1,000 days). Thigh injuries were most common (7.4/100 players/season), while lumbar spine issues had the highest prevalence (1.3% daily unavailability). Overall, 7.5% of players were unavailable due to injury, with trends consistent over nine seasons
Schaefer et al., 2020 [29]	Cross-sectional observational study	60 junior male fast bowlers (12–18 years) from district and zone-level teams in New South Wales, Australia	Comparing biomechanical differences among front-on, semi-open, and mixed bowling actions	Trunk, pelvis, lumbar spine angles, and GRF loading rates during back-foot and front-foot contact	Front-on bowlers had greater trunk rotation, mixed action bowlers showed more lumbar motion, and semi-open bowlers had lower GRF loading rates. No significant differences in overall joint loading
Always et al., 2020 [30]	Prospective cohort study	45 elite male fast bowlers aged 18–19	Examining lumbar spine joint angles to determine their role in LBSIs	Upper and lower lumbar spine joint angles during fast bowling	No significant lumbar spine angle differences between injured and non-injured bowlers, but a trend toward greater upper lumbar extension in injured bowlers suggests potential increased stress on the neural arch. Other factors may contribute more to LBSI risk
Warren et al., 2019 [31]	Prospective cohort study	Elite female T20 cricketers (16–38 years, mean 23.4 ± 4.8)	Injury incidence and prevalence over two years of a professional T20 tournament	Injury classification, affected region, skill group, injury mechanism, and activity at occurrence	Shoulder, lower back, and knee were the most common injury sites. Hand, head/face (concussions), and thigh injuries had the highest time-loss. Catching caused the most time-loss, while throwing led to the highest shoulder injury incidence. Gradual onset injuries were frequent, emphasizing workload management. Trauma training and pitch-side support were key priorities due to high fielding impact injuries
Gamage et al., 2019 [32]	Prospective	573 Sri Lankan junior cricketers (U-15 and U-17) from Division-1 teams in competitive cricket	Incidence and nature of match injuries	Injury site, type, mechanism, match-time loss (MTL) vs. non-MTL injuries, and position-specific injury rates	Match injury incidence rate (IIR) was 28.0 per 100 match-player-days. Fielders had the highest injury rate (46%, IIR = 12.9), mainly knee/elbow abrasions. Batters faced facial injuries (IIR = 7.1), emphasizing helmet safety. Bowlers suffered lower limb and back strains (IIR = 5.7). Fielder injuries were mostly non-MTL, while batter injuries had higher severity
Always et al., 2019 [33]	Retrospective epidemiological study	368 elite English County Cricket fast bowlers	Incidence, prevalence, and workload-related risk factors for lumbar stress fractures	Match workload, seasonal variation, and peak bowling loads	57 lumbar stress fractures were recorded, peaking in July and September. Match incidence was 0.16 per 10,000 deliveries, with an annual incidence of 2.46 per 100 fast bowlers. A peak 7-day workload of over 234 deliveries increased the risk 11-fold compared to fewer than 197 deliveries. Young bowlers may be more vulnerable due to spinal immaturity and early/late-season overuse
Krishna et al., 2018 [34]	Cross-sectional	22 male state-level Indian fast bowlers (18–30 years) competing in state-level cricket	Identifying injury risk in Indian fast bowlers through biomechanical analysis	Stride length, lateral trunk flexion at ball release, knee flexion at front-foot contact, and peak vertical GRFs	All bowlers had suboptimal stride length, 41% showed excessive lateral trunk flexion, 45% had high peak vertical GRFs, 45% had low knee flexion at front foot contact, and 77% had an acceptable bowling action
Schaefer et al., 2018 [35]	Experimental study	25 junior male fast bowlers (12–19 years) from district/zone-level teams	Investigated workload-induced fatigue effects on biomechanics and injury risk	Examined changes in performance, joint kinematics, kinetics, GRFs, and movement variability across overs	No significant biomechanical changes or injury risk increase were observed, despite a slight, non-significant drop in ball speed
Sathya et al., 2017 [36]	Cross-sectional survey	125 male club-level cricket players	Prevalence of musculoskeletal problems in cricket players	Player role (all-rounder, bowler, batsman) and injury type	61% of players reported musculoskeletal issues, mainly in the lower back, ankle, and knee. All-rounders had the highest injury rate (70%), followed by bowlers (60%) and batsmen (42%). Strains (42%) and sprains (26%) were the most common
Bayne et al., 2016 [37]	Prospective study	25 injury-free male fast bowlers, aged 14–19 years	Identifying biomechanical and physical risk factors for low back injury	Bowling kinematics, lumbar load, muscle endurance, and movement control	Injured bowlers showed reduced hip flexion, increased pelvis rotation, thorax lateral flexion, higher lumbar moments, and lower back extensor endurance
Orchard et al., 2015 [7]	Retrospective injury surveillance	Elite senior male Australian cricketers	Injury incidence and prevalence in different cricket formats (T20, 50-over, first-class)	Match format, player position (especially fast bowlers), and workload	Match injury incidence was highest in 50-over cricket (155 injuries/1,000 days). Annual injury rate was 64 injuries/100 players, with a 12.5% prevalence—fast bowlers had the highest (20.6%). Hamstring strains were most common (8.7/100 players), while lumbar stress fractures caused the most missed time (15%). Increased T20 cricket correlated with more hamstring strains, while lumbar stress fractures remained a major concern due to high workloads. International players faced higher injury rates due to continuous play
Blanch et al., 2015 [38]	Retrospective analysis	215 fast bowlers, categorized into five age groups	Investigating injury incidence, severity, tissue type, and the influence of age on injury risk	Age, tissue type (bone vs. tendon), and injury severity	Younger bowlers (<22) had a 3.7–6.7 times higher risk of bone stress injuries, while older bowlers (>31) had a 2.2–2.7 times higher risk of tendon injuries. Injury type varied significantly with age
Orchard et al., 2015 [39]	Prospective cohort study	235 fast bowlers, covering 14,600 player innings	Investigated how bowling workload influences bone stress, tendon, muscle strain, and joint injuries	Acute, medium-term (3-month), previous season, and career workloads	Tendon injuries: High acute and previous season workload increases risk; medium-term workload offers protection. Bone stress injuries: Higher risk with high medium-term but low career workload. Joint injuries: Increased risk with high previous season and career workload. Muscle injuries: Minimal correlation; high previous season workload may be protective
Ranson et al., 2013 [40]	Injury surveillance study	Multiple international cricket teams (elite-level players)	Injury incidence, prevalence, and patterns during a major international tournament	Injury type, affected body regions, player roles, and match workload	Injury incidence: 3.7 injuries/100 player-days (0.7 time-loss, 3.0 non-time-loss). Match time-loss injuries: 20.1/1000 player-days. Bowling injuries: 3.3/100 bowling days; batting injuries: 2.2/10,000 balls faced. Common injuries: Thigh muscle strains and medical illnesses. Injury prevalence: ~5% for fast bowlers, slow bowlers, and batters. Highest time-loss activity: Bowling delivery stride
Crewe et al., 2013 [41]	Experimental study	Nine asymptomatic right-arm fast bowlers (mean age: 16.9 years) from district/state junior squads	Evaluating how different BSP methods affect calculated lumbopelvic forces and moments during fast bowling	BSP variability affects lumbar kinetics, potentially leading to inaccuracies in kinetic calculations	BSP methods showed no impact on peak lumbo-pelvic forces, but vertebral-level segmentation led to higher flexion and lateral flexion moments, affecting study comparisons
Crewe et al., 2013b [42]	Biomechanical study	40 male adolescent fast bowlers (16.2 years); 23 completed the full 8-over spell	Examined lumbar loading during bowling and its association with shoulder counter-rotation, front knee kinematics, and ball release speed	Examined shoulder counter-rotation, front knee flexion angle, and ball release speed	Lumbar loading remained stable over the 8-over spell. Higher ball speed, straighter front knee, and greater shoulder counter-rotation increased shear forces and rotational moments
Stretch et al., 2012 [43]	Injury surveillance study	1,292 players from 16 provincial U15, U17, and U18 teams in national tournaments	Assessing injury incidence, sites, causes, recurrence, and risk factors	Examined injury patterns by playing format, mechanism (bowling, fielding), and type (acute, chronic, recurrent)	28% of players sustained 425 injuries, primarily in the lower (46%) and upper limbs (35%), with most occurring during 1-day matches (31%) and practices (27%). Bowling (45%) and fielding (33%) were the main causes. Common injuries included lumbar muscle strains (42), hamstring strains (18), spondylolisthesis (17), and ankle sprains (17). Recurrent injuries accounted for 24% from the previous season and 46% within the same season. U15 players had mostly minor injuries, U17 had the most lumbar strains (23), and U18 suffered more severe injuries, with 60% requiring 8+ days of recovery
Crewe et al., 2011 [44]	Experimental study	10 male right-arm fast bowlers (16.8 ± 1.3 years) from district/state junior squads	Investigating SCR’s impact on lumbar kinematics, kinetics, and injury risk in fast bowling	Examined SCR, lumbar ROM, peak lumbar moments, and GRF during the delivery stride	Peak vertical GRF: 5.6 times body weight. SCR strongly correlated with lumbar rotation ROM and moderately with lateral flexion ROM. Lumbar flexion moments linked to increased lumbopelvic rotation
Stretch et al., 2011 [45]	Prospective injury surveillance	Elite cricketers from 4 provincial teams	Examined injury incidence, location, causes, and recurrence in elite cricketers	Compared match vs. training injury rates, injury sites, mechanisms (bowling, fielding, batting, overuse, impact), and recurrence patterns	180 injuries over two seasons; incidence: 30/10,000 hours (matches: 74; training:15). Lower limbs (44%) and back (19%) were the most affected. Common injuries: strains, tendinopathy, hematomas. Fast bowlers were most at risk, with acute injuries (78%) dominating. Main causes: delivery stride (19%), overbowling (7%), batting impact (11%), and fielding slides (6%)
Stuelcken et al., 2010 [46]	Cross-sectional retrospective study	26 elite female fast bowlers (mean age 22.5 ± 4.5 years) from the Australian women’s cricket	Examined thorax-pelvis movement patterns in relation to LBP history	Classified bowling techniques and analyzed shoulder counterrotation, pelvis-shoulder separation angles, and thorax-pelvis lateral flexion	54% had LBP history; 73% used a mixed action, but no direct link to LBP. Greater thorax lateral flexion was found in LBP bowlers (p = 0.004). No significant differences in thorax-pelvis alignment at front foot contact. Shoulder counterrotation was not a strong predictor. Excessive lateral flexion may contribute to LBP risk
Ferdinands et al., 2010 [47]	Cross-sectional	13 elite fast bowlers (mean age 17.4 ± 1.9) from the Cricket NSW development squad	Examined bowling kinematics and lumbar spine loading to enhance injury risk assessment	Analyzed shoulder counter-rotation, pelvic-shoulder separation, thoracic and pelvic motion, knee flexion, stride angle, and bowling hand velocity	Significant lumbar spine forces were linked to kinematic factors like shoulder counter-rotation and stride angle, while others (e.g., stride length) showed no correlation. Findings support the link between kinematics and spinal loading but highlight the need for longitudinal studies and MRI-based injury assessment
Middleton et al., 2009 [48]	Experimental study	17 male first-grade fast bowlers (mean age: 20.9 years) from Western Australia	Examined the relationship between ball release velocity and shoulder alignment, knee flexion, trunk lateral flexion, and counter-rotation	Examined the impact of SA, KF, TLF, and CR on ball velocity and their link to lower back injury risk	Faster deliveries (32.46 m/s) differed significantly from normal ones (31.31 m/s, p < 0.001). No link was found between CR, KF, and ball speed. Less TLF (“falling away”) correlated with higher velocity. SA increased with speed but raised lower back injury risk. TLF and SA explained only 11% of ball velocity variance, making them weak predictors
Ranson et al., 2008 [3]	Cross-sectional	48 professional male fast bowlers (mean age 22 ± 3 years) from English County Cricket, recognized by the ECB	Investigated lower back injuries concerning bowling technique, trunk kinematics, lumbar MRI findings, and muscle asymmetry	Bowling action type, trunk kinematics, lumbar MRI findings, and muscle asymmetry	35% sustained lower back injuries; 25% had acute lumbar stress injuries, missing 106 days on average. No link between mixed action and injury. Reduced contralateral side-flexion ROM (p = 0.03) and MRI-detected stress changes (p = 0.001) were associated with injury. Muscle asymmetry showed no clear impact. Regular MRI screening may aid in early detection
Ranson et al., 2008b [49]	Cross-sectional	50 professional male fast bowlers (mean age: 23) from English County Cricket clubs	Analyzed lower trunk movements during delivery stride and their link to bowling action type and injury risk	Examined shoulder counter-rotation, alignment, and lower trunk kinematics (extension, side-flexion, axial rotation)	78% of bowlers used a mixed action, with no front-on actions observed. Shoulder counter-rotation correlated strongly with shoulder alignment but weakly with trunk rotation. No significant differences in lower trunk kinematics between action types. Early front foot contact with trunk extension, ipsilateral rotation, and extreme contralateral side-flexion may contribute to lumbar stress injuries. Bowling action classifications may not fully explain injury risks, warranting further research

Table 2 categorizes key findings into thematic areas such as injury prevalence, anatomical regions affected, biomechanical contributors, bone mineral density (BMD) adaptations, workload influences, and gender-based or positional differences.

**Table 2:** Synthesis of articles, including categories, key findings, and corresponding references. The included studies were synthesized based on study design, participant characteristics, risk factors analyzed, and key findings related to injury epidemiology, biomechanics, and risk assessment in cricket fast bowlers. A comprehensive synthesis approach was used, where similar outcomes were grouped to establish trends and key determinants of injury occurrence and performance implications.

Category	Key findings	References
Injury prevalence	Fast bowlers had the highest injury rates across all studies. T20 cricket showed the highest injury incidence. Match-related injuries were more frequent than training injuries	[7,19,22]
Common injury sites	Lumbar spine (17–42%) – stress fractures, muscle strains; Lower limbs (30–45%) – knee, thigh, and ankle injuries; Shoulder (15–22%) – rotator cuff inflammation, impingement	[15,19,27,28]
Injury mechanism	Bowling-related injuries accounted for over 50% of all injuries. Acute injuries (68%) were more frequent than overuse injuries (32%)	[19]
Biomechanical risk factors	Shoulder counter-rotation >25°, excessive lateral trunk flexion, and reduced knee flexion at front-foot contact increased lumbar stress. Greater ankle dorsiflexion reduced lumbar stress injury risk. Poor lumbopelvic control predicted 88% of lumbar injuries	[17,23,34,41]
Bone mineral density and skeletal adaptation	Fast bowlers had greater lumbar bone mineral density than other cricketers, with pronounced asymmetry at L3–L4. Younger bowlers (<22 years) had a 3.7–6.7 times higher risk of bone stress injuries, while older bowlers (>31 years) had more tendon injuries. High workloads without proper rest led to early bone stress injuries	[16,18,20,38]
Bowling workload and injury risk	Peak 7-day bowling workloads (>234 deliveries) increased lumbar stress fracture risk 11-fold. Recent high bowling frequency was a stronger injury predictor than long-term workload. Prior lumbar injury was the strongest predictor of recurrence (63% reinjury rate, median return time: 161 days	[21,33]
Gender and positional differences	Female fast bowlers had higher lumbar injury rates, while batters had more overall injuries. Male bowlers showed higher bone mineral density asymmetry. Injured females bowled more in the last four weeks, while injured males had lower 12-month workloads	[15,16,21]

Table 3 presents a focused summary of biomechanical, workload, anatomical, technique-related, and injury-history variables associated specifically with lumbar stress injuries in cricket fast bowlers. Each category includes individual variables (e.g., shoulder counter-rotation, lateral trunk flexion, bowling workload, BMD asymmetry) along with their documented impact on lumbar stress injury risk

**Table 3:** Biomechanical, workload, anatomical, technique, and injury history variables linked to lumbar stress injuries.

Category	Variable	Impact on Lumbar Stress Injuries	References
Biomechanical variables	Shoulder counter-rotation	>25° increases spinal loading and stress fractures	[34,41]
	Trunk lateral flexion	Excessive flexion leads to higher lumbar loads and increased stress	[17]
	Knee flexion at Front-foot contact	Reduced flexion at impact increases lower back strain	[23]
	Pelvic control	Poor control predicts 88% of lumbar injuries	[23]
	Ankle dorsiflexion	Greater dorsiflexion reduces lumbar stress injury risk	[17]
Workload variables	Peak bowling workload	High workloads (>234 deliveries) increase lumbar stress fractures	[21]
	Recent high bowling frequency	Stronger injury predictor than long-term workload	[21]
Anatomical variables	Bone mineral density	Asymmetry at L3–L4 increases the risk of lumbar stress injuries	[16,18]
	Age factor	Younger bowlers have a 3.7–6.7 times higher risk of stress fractures	[20,38]
Technique-related variables	Front-foot impact mechanics	Reduced knee flexion and excessive lateral flexion increase lumbar stress	[34,42]
Injury history variables	Prior lumbar injury	Strongest predictor of recurrence (63% reinjury rate)	[21]

Injury Prevalence and Patterns

Fast bowlers experience the highest injury rates across all playing levels. Injury incidence in fast bowlers was significantly higher compared to other cricketing roles [7,9,22]. The T20 format exhibited the highest injury incidence, with domestic leagues reporting 74.5 injuries per 10,000 hours and international leagues 144.2 per 10,000 hours [22]. Bowling-related injuries accounted for over 50% of all injuries in elite-level cricketers [19].

Most commonly affected body regions included the lumbar spine (17-42%), with stress fractures and muscle strains [19,21,27]; lower limbs (knee, thigh, ankle) (30-45%), with hamstring strains and quadriceps injuries [15,28]; shoulder (15-22%), with Rotator cuff inflammation and impingement syndromes [27,31].

Match injuries outnumbered training injuries. Studies reported that over 50% of injuries occurred during matches, with time-loss injuries more frequent in high-intensity formats [7,28]. Acute injuries were twice as common as overuse injuries [28].

Biomechanical and Kinematic Risk Factors

Key biomechanical contributors to lumbar stress injuries: Shoulder counter-rotation (>25°) and excessive lateral trunk flexion were linked to high lumbar stress [34,41]. Greater ankle dorsiflexion (>30°) reduced lumbar stress injury risk, highlighting its shock-absorbing role [17]. Lower knee flexion (<30°) at front-foot contact increased ground reaction forces, elevating lumbar and knee injury risks [34].

Lumbopelvic control and lumbar spine motion were strong predictors of injury: Poor lumbopelvic control increased the risk of lumbar stress fractures by 88% [23]. Excessive lumbar extension at front-foot impact placed additional stress on the neural arch [30]. Inter-individual variability in spinal kinematics was high, reinforcing the importance of individualized assessments [25].

Bone Mineral Density and Skeletal Adaptations

Fast bowlers displayed greater lumbar BMD than other cricketing roles, with adaptation varying by age and workload [16,18]. BMD gains were most pronounced at L3-L4, with an asymmetry of up to 15% on the contralateral side [18]. Younger bowlers (<22 years) had a 3.7-6.7 times higher risk of bone stress injuries, while older bowlers (>31 years) were more prone to tendon-related injuries [38]. Excessive bowling loads without proper rest led to early bone stress injuries, reinforcing the importance of periodized training [20].

Workload and Injury Risk

High bowling workloads significantly increased lumbar stress injury risk: Bowlers who delivered over 234 balls in a seven-day period had an 11-fold higher risk of lumbar stress fractures [33]. Recent high bowling frequency (last four weeks) was a stronger predictor of injury than cumulative workload over 12 months [21]. Previous lumbar injury was the strongest predictor of recurrence, with a 63% reinjury rate and a median return time of 161 days [21].

Positional and Gender-Based Comparisons

Elite female cricketers exhibited different injury profiles compared to male bowlers. English female cricketers sustained higher lumbar injury rates, while Indian female batters had higher overall injury incidence [15]. Fast bowlers exhibited greater lumbar BMD asymmetry, reinforcing the positional impact forces [16]. Injured female bowlers had higher recent bowling volumes, whereas injured male bowlers exhibited lower long-term workloads [21].

Discussion

Significant advancements in sports biomechanics have greatly enhanced our understanding of the relationship between injury mechanisms and performance in cricket fast bowling. The evolution of biomechanics in cricket fast bowling has revolutionized the understanding of injury mechanisms and performance optimization. Over the past two decades, advances in biomechanical analysis have shifted from basic observational methods to sophisticated assessments of movement patterns, joint mechanics, and load distribution. This transformation has been driven by improved technology and deeper insights into the complex interactions between kinematics (movement) and kinetics (forces).

The introduction of novel technologies such as 3D motion capture (MoCap) and force plate analysis is now considered the gold standard for early injury detection, biomechanical screening, and performance monitoring [11,49]. These tools enable detailed assessment of both kinetic and kinematic variables, helping identify high-risk injury patterns such as excessive trunk rotation, poor lumbopelvic control, abnormal hip-shoulder separation angles, and higher vertical ground reaction forces [44]. The application of biomechanics is crucial in optimizing individualized technique modification and managing training loads, both of which are essential for reducing injury risk and enhancing long-term athletic performance [46]. As such, biomechanics has become an indispensable tool in the scientific development and support of fast bowlers at all levels of play.

Biomechanical Risk Factors

Biomechanical factors play a crucial role in lumbar stress injury development. Limited range of motion, stride length variations, and excessive lateral trunk flexion contribute to increased spinal stress [17,48]. Greater ankle dorsiflexion appears protective against lumbar stress injury, while excessive counter-rotation and lateral flexion elevate spinal loading [41,49]. Variability in spinal kinematics further suggests that individualized biomechanical assessments could help reduce injury risks [25].

Recovery and Workload Management

Recovery from lumbar stress injury is prolonged, with a median return-to-play time of 161 days and high reinjury rates [21]. Stress fractures result in significant time loss, affecting player availability and long-term career prospects [19]. Effective workload management is crucial, as excessive acute or chronic bowling loads heighten injury risk. A peak seven-day workload exceeding 234 deliveries raises lumbar stress injury risk 11-fold, while moderate workload exposure appears protective [32,38]. Injury rates are also higher in T20 formats due to intense match demands and reduced recovery time [22,28].

Gender-Specific Considerations

In female cricketers, injury patterns differ from those in males, with wrist, hand, and thigh injuries being more common [15,31]. Additionally, fast bowlers exhibit asymmetric BMD adaptations, with higher lumbar BMD on the contralateral side due to high-impact forces. While this may provide osteogenic benefits, it also increases the risk of asymmetric loading injuries [16].

Future research directions

Further investigation is needed to refine injury prevention strategies. While studies have established associations between shoulder counter-rotation, lumbar kinetics, and spinal loading [40-46], longitudinal research incorporating MRI-based assessments is essential to validate these findings and develop targeted interventions.

Limitations

This study has several limitations. Variability in study designs, data collection methods, and injury diagnostic criteria may affect the consistency of findings. The predominance of cross-sectional studies limits causal inferences, and the focus on elite male fast bowlers reduces the applicability of results to female and junior athletes. Inconsistencies in injury classification and reliance on self-reported symptoms may contribute to underdiagnosis. Additionally, psychological, nutritional, and genetic factors influencing injury risk remain underexplored.

While biomechanical and workload-related risks are well-documented, evidence on targeted prevention and rehabilitation strategies is still limited. This study does not validate the quality of the included research articles but focuses solely on reported outcomes, which may introduce selection bias. Furthermore, subjective biases from participants and researchers could impact the study’s findings. To enhance the accuracy of injury assessment, future research should incorporate longitudinal follow-up studies using gold-standard diagnostic tools such as MRI, CT scans, dual-energy X-ray absorptiometry, and motion capture technology. Standardized assessments and prospective cohort studies are essential to bridging existing knowledge gaps and improving injury prevention strategies.

## Conclusions

Lumbar stress injuries in fast bowlers result from a multifactorial interaction of biomechanical, workload, anatomical, and technique-related variables. Excessive shoulder counter-rotation, lateral trunk flexion, poor lumbopelvic control, and high ground reaction forces are key contributors that elevate lumbar loading, while factors such as optimal ankle dorsiflexion may provide protective benefits. Acute workload spikes and individual variability in spinal motion further intensify injury risk, especially in younger bowlers. Overall, effective prevention requires early identification of high-risk movement patterns through biomechanical screening, systematic workload monitoring, and targeted strengthening of the lumbopelvic-hip complex. By integrating personalized biomechanical insights into training and rehabilitation, fast bowlers can optimize performance while reducing injury risk, underscoring the essential role of biomechanics in supporting long, healthy sporting careers.
